# Supplementary material for: Brief segments of neurophysiological activity enable individual differentiation
Source: Nat Commun. 2021 Sep 29;12:5713. doi: 10.1038/s41467-021-25895-8 (PMC8481307; doi:10.1038/s41467-021-25895-8)
Supplement: Supplementary file 1 — Supplementary Information [file 41467_2021_25895_MOESM1_ESM.pdf]

Supplementary Information

Supplementary Notes

MEG fingerprinting is robust against sample demographics

The OMEGA data repository contains 158 participants, with a subset (N=47) scanned at multiple occasions several days apart. OMEGA consists essentially of data from healthy controls with a 18-73-year age span (SD=14.7 years; Supplementary Table 1).

One potential confound that could have inflated our ability to fingerprint individuals is the heterogeneity introduced by both healthy and clinical populations in the OMEGA cohort. To address this concern, we ran a secondary analysis where we performed the fingerprinting procedures described in the manuscript with only healthy controls (N=130). The results, reported in Supplementary table 2, demonstrated that fingerprinting performances were not biased by the patients/controls heterogeneity of the OMEGA sample. We observed a decrease of less than 1% in performance relative to fingerprinting from the entire cohort. Further, there was no clear relationship between differentiability** and demographics (Supplementary Figure 1)., using connectome (age: r= 0.08, p = 0.2; gender: t= -0.27, p = 0.7; handedness: t= -0.51, p = 0.6; clinical status: t= -0.87, p = 0.3; two-tailed) and spectral fingerprinting (age: r= 0.10, p = 0.1; gender: t= 0.62, p = 0.5; handedness: t= 0.13, p = 0.8; clinical status: t= 0.84, p = 0.3; two-tailed).

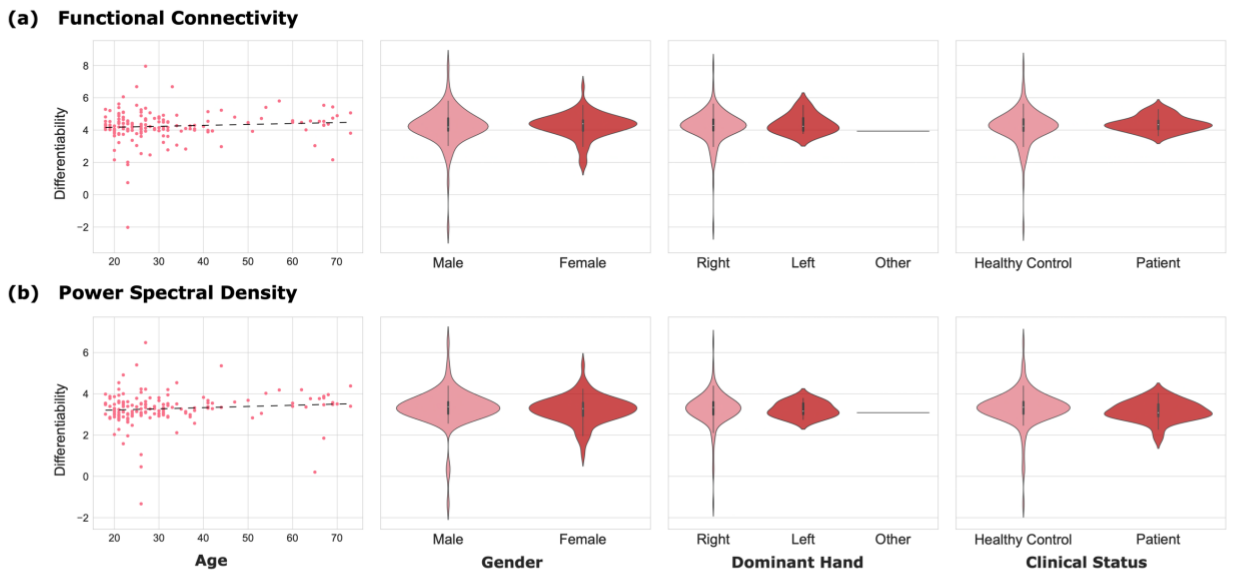

Supplementary Figure 1: Differentiability is not associated with demographics

The plots depict demographic variables and corresponding differentiability scores across both **(a)** connectome and **(b)** spectral broadband within-session fingerprinting (n = 158). Demographic variables included age, biological sex, dominant hand, and healthy vs. patient categories. **(a)** There was no clear relationship between age, biological sex, dominant hand, and healthy vs. patient categories and differentiability for broadband within-session connectome fingerprinting. **(b)** There was no clear relationship between age, biological sex, dominant hand, and healthy vs. patient

** Please note that differentiability may also be referred to as self-identifiability (I_{self}) in our analysis scripts

categories and differentiability for broadband within-session connectome fingerprinting. Differences in demographics did not drive differentiability. The center of the boxplot depicts the median (Q2), the box extends from the Q1 to Q3 quartile values of the data, and the whiskers extend to show the range of the data (i.e., the farthest datapoint within the 1.5 * IQR (IQR = Q3 - Q1) interval). Source data are provided as a Source Data file.

Acquisition parameters did not affect both fingerprinting performances (Supplementary Figure 2). Participants with longer recordings (i.e., more data) were not more differentiable (connectome: r= -0.02, p = 0.7; spectral: r= 0.02, p = 0.8). This observation is consistent with the shortened fingerprinting results, which demonstrate individuals were differentiable from shorter 30-second recordings (see below).

Taken together, these supplemental results demonstrate that MEG fingerprinting is robust against data artifacts, heterogeneous sample demographics, and acquisition parameters.

	Within-session data	Between-session data
Age	31.9 ± 14.7	26.7 ± 11.6
Gender	77 Females	24 Females
Dominant Hand	147 Right, 8 Left, 1 Other	44 Right, 3 Left
Clinical Status	130 Healthy Controls<br>22 ADHD<br>6 Chronic Pain	25 Healthy Controls<br>22 ADHD

Supplementary table 1: OMEGA participant demographics

Demographic variables summarized for both subsets of the OMEGA data repository.

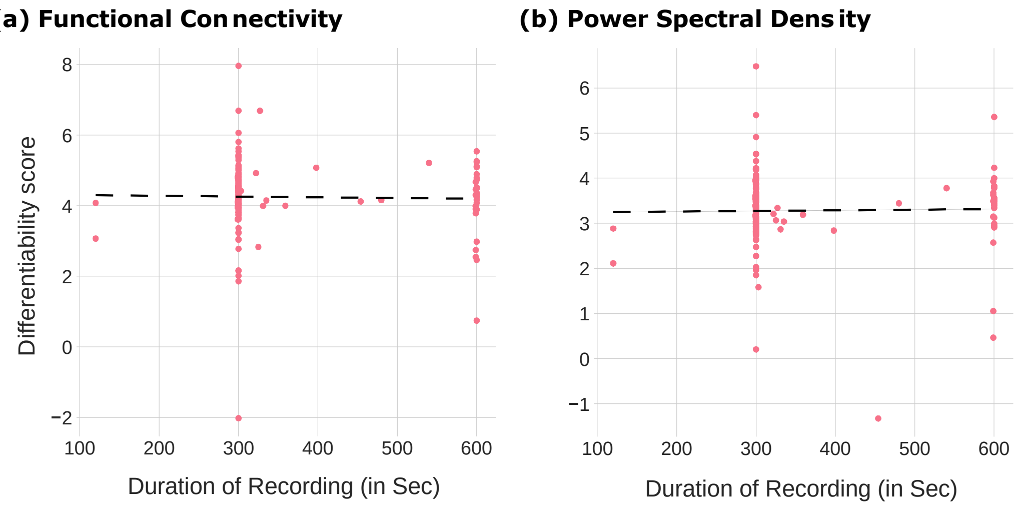

Supplementary Figure 2: Recording duration did not affect differentiability
Scatter plots of differentiability vs. duration of data collections, for the broadband within-session challenge. There was no clear relationship between differentiability and the duration of the MEG recordings across participants. Source data are provided as a Source Data file.

	All Participants		Only Healthy Controls	
	Dataset 1 to Dataset 2	Dataset 2 to Dataset 1	Dataset 1 to Dataset 2	Dataset 2 to Dataset 1
Connectome	94.9%	94.3%	93.8%	93.0%
Spectral	96.2%	96.2%	95.3%	95.3%

Supplementary table 2. Fingerprinting performances of healthy controls
Differentiation performances of connectome and spectral broadband within-session fingerprinting obtained from for the entire repository (healthy controls and patients), and from healthy participants only. Each column reports fingerprinting performances from dataset 1 to dataset 2 and vice-versa (see Figure 1 and Methods for details). Overall, differentiation accuracy decreased slightly by ~0.9% when comprising healthy participants only. Consistent with our findings reported in Supplementary Figure 2, clinical status did not play a major role in the differentiation of individuals.

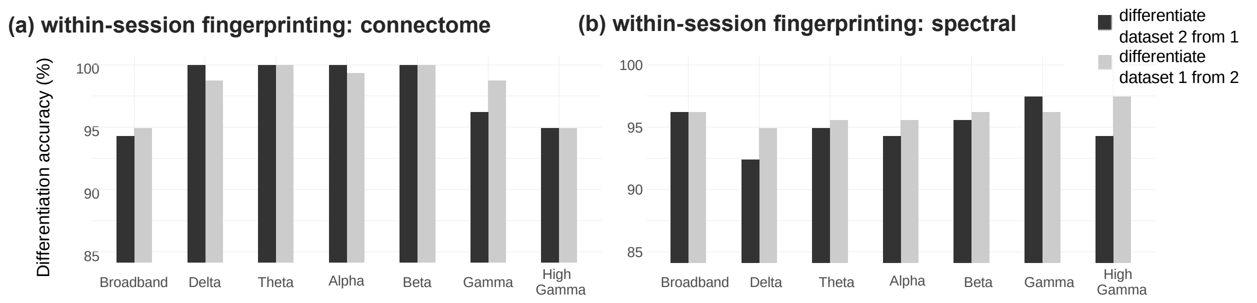

Supplementary Figure 3: Differentiation accuracy from within-session datasets
Results from MEG within-session fingerprinting. Differentiation accuracy for **(a)** connectome and **(b)** spectral fingerprinting (broadband and narrowband data). The accuracy scores are reported for differentiation from dataset 1 to dataset 2 and vice-versa, as explained in Methods. Source data are provided as a Source Data file.

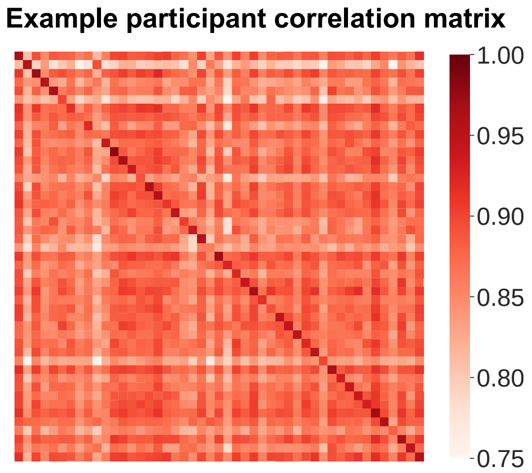

Supplemenatry Figure 4: Example participant correlation matrix for fingerprinting
Exemplar participant correlation matrix derived from between-session data used for fingerprinting. The study-identity of participants was determined by the highest correlation statistics taken across rows (e.g., to differentiate dataset-2 from dataset-1) or columns (to differentiate dataset-1 from dataset-2).

Data reduction from principal component analysis does not improve MEG fingerprinting substantially

Amico and Goñi (*1*) previously reported improvements to participant differentiation when using data reduction techniques prior to fingerprinting, using e.g., principal component analysis (PCA). We reproduced their approach, using PCA to reduce the dimensionality of the connectome and spectral feature spaces prior to fingerprinting. Our results provided little support to PCA reconstruction improving differentiation accuracy, as shown Supplementary Figure 5 and in Supplementary table 3. PCA increased differentiability by less than 1.5%. Data reduction had limited beneficial impact possibly because of high fingerprinting performances at baseline (without data reduction). We also emphasize that we conducted MEG source time series extraction via a PCA of all local time series within each parcel. It is therefore likely that this dimension reduction procedure contributed to improve signal-to-noise ratio and limited the impact of subsequent PCA of features.

	Original (un-reconstructed)		PCA Reconstructed	
	Dataset 1 to Dataset 2	Dataset 2 to Dataset 1	Dataset 1 to Dataset 2	Dataset 2 to Dataset 1
Connectome	94.9%	94.3%	96.2%	96.2%
Spectral	96.2%	96.2%	96.2%	96.2%

Supplementary table 3: Limited contribution of data reduction from principal component analysis to MEG fingerprinting.
Performances in differentiation accuracy for connectome and spectral broadband within-session fingerprinting, for both original and PCA-reconstructed data (1). PCA data reduction improved connectome fingerprinting performances only slightly (about 2%). It had virtually no effect on spectral fingerprinting performances.

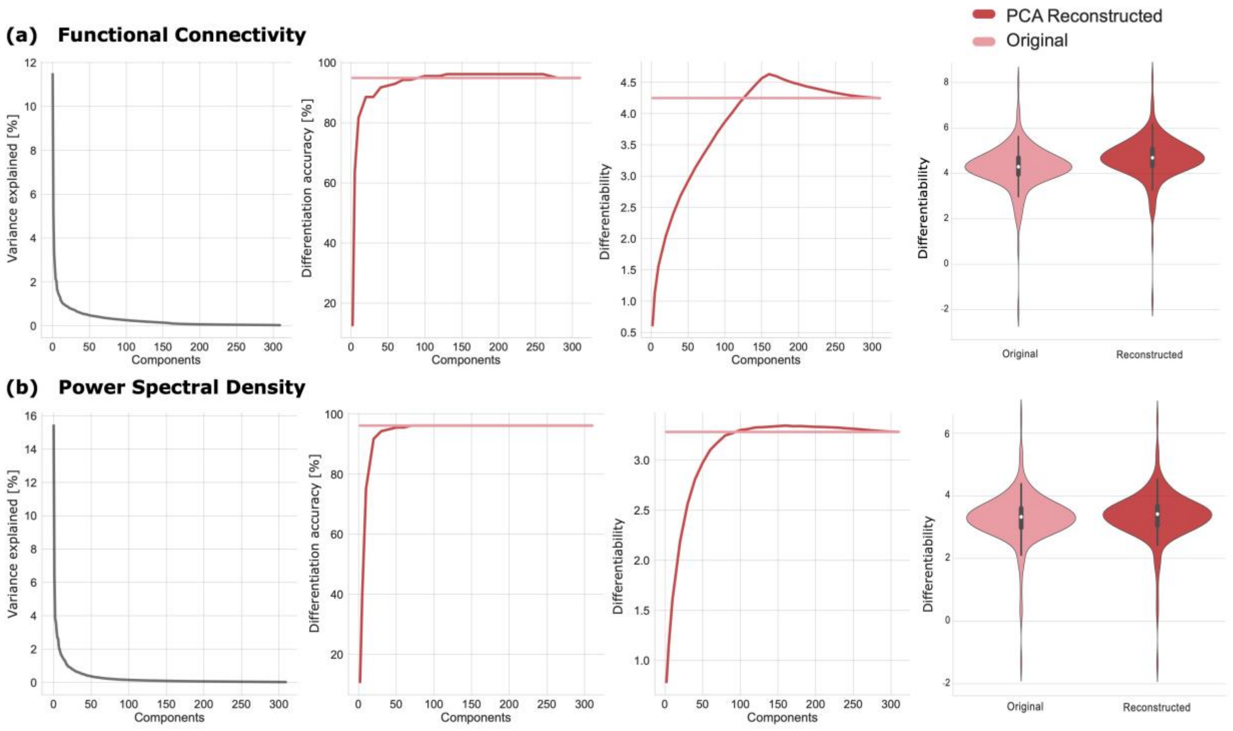

Supplementary Figure 5: Limited benefit of PCA reconstruction to differentiation accuracy
PCA reconstruction as proposed by Amico and Goñi (2018) had limited effect on **(a)** connectome and **(b)** spectral within-session fingerprinting (n= 158). The original results (Figure 2) are plotted against PCA-reconstructed results. From left to right, plots show *i)* PCA components plotted vs. their respective fractions of signal variance explained, *ii)* differentiation accuracy across PCA components, *iii)* average differentiability scores across PCA components, and *iv)* violin plots of differentiability scores before and after PCA reconstruction for both **(a)** connectome and **(b)** spectral fingerprinting. Overall, PCA reconstruction did not substantially improve differentiation accuracy. The center of the boxplot depicts the median (Q2), the box extends from the Q1 to Q3 quartile values of the data, and the whiskers extend to show the range of the data (i.e., the farthest datapoint within the 1.5 * IQR (IQR = Q3 - Q1) interval). Source data are provided as a Source Data file.

Fingerprinting with 30-second data segments

We challenged MEG fingerprinting using short 30-second data segments (i.e., shortened within-session fingerprinting). We epoched participants' MEG recordings into three datasets of 30 second, where the first dataset was the first 30 seconds of the recording after having removed the

initial five seconds, the second dataset was the 30 seconds immediately following the first dataset, and the last dataset was the last 30-second segment of the recording after having removed the last ten seconds (see Figure 1). Cropping the initial and last few seconds from recordings excluded edge, filtering, and other session artifacts. The lengths of the short datasets and epochs were determined from the participant with the shortest available recording. This procedure yielded three data segments for fingerprinting purposes via 6 possible dataset pairs (i.e., dataset 1 and 2; dataset 2 and 3; and dataset 1 and 3 and vice-versa). Results for all possible combinations of datasets are reported in Supplementary Figure 6.

Connectome fingerprinting successfully differentiated individuals across all possible combinations of datasets (Supplementary Figure 6). Fingerprinting from recordings collected closer in time (e.g., dataset-1 and dataset-2) outperformed differentiation from datasets collected further apart in time (e.g., between dataset-1 and dataset-3). Overall, spectral fingerprinting yielded lower differentiation accuracy than connectome fingerprinting, in particular from datasets further apart in time.

In a similar fashion, we challenged MEG fingerprinting using short 30-second data segments from different sessions (i.e., between-session fingerprinting). This yielded 6 epochs of data for fingerprinting (i.e., three from both the first and second recording, see Figure 1a). Fingerprinting results averaged across all possible data pairs are reported Figure 3c. Connectome fingerprinting performances were greater than those from spectral fingerprinting. Differentiation from slower frequency data components performed worse in comparison to higher bands – see main article body for a discussion.

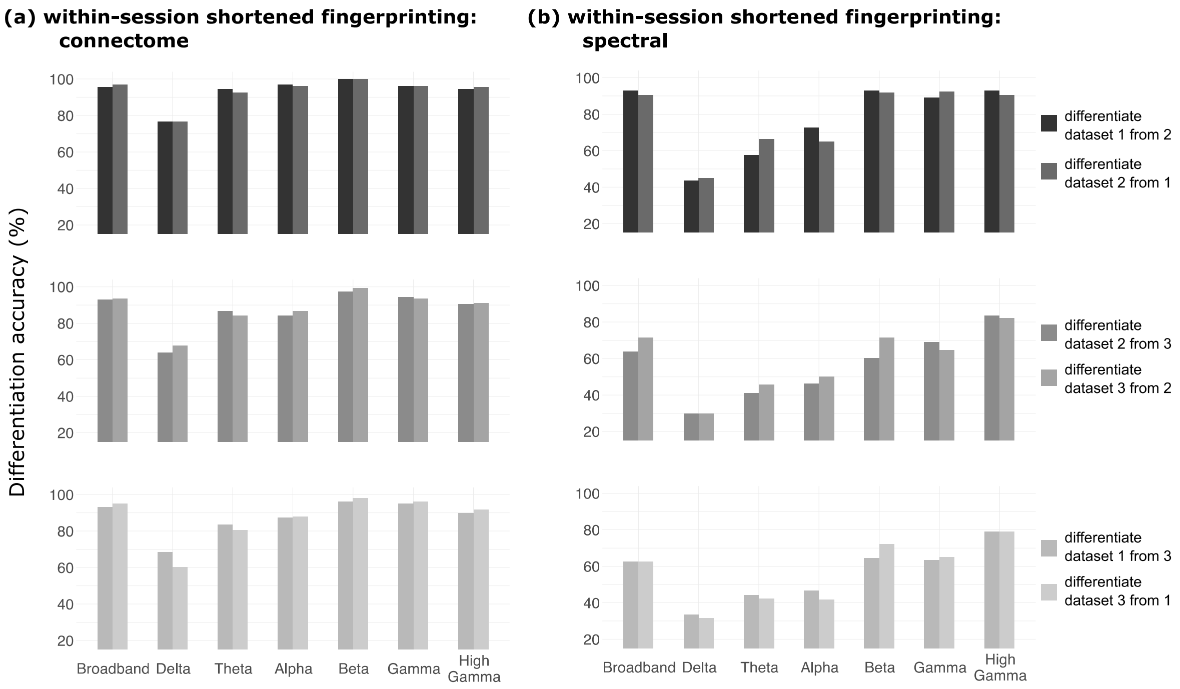

Supplementary Figure 6: Differentiation accuracy from shortened within-session datasets

Differentiation results from shortened within-session datasets (30 seconds) for (a) connectome and (b) spectral broadband and narrowband fingerprinting. The accuracy scores are reported for differentiation from all possible combinations of datasets, (i.e., dataset 1 to predict dataset 2, dataset 3 to predict dataset 2, etc.; see Methods for details). Differentiation accuracy increased as datasets were proximal in time (i.e., fingerprinting accuracy for dataset 1 to dataset 2 was greater than for dataset 1 to dataset 3). Source data are provided as a Source Data file.

Fingerprinting across recording sessions

We also report fingerprinting accuracy performances from all possible pairs of datasets for the between-session fingerprinting challenge in Supplementary Figure 7. Overall, spectral fingerprinting outperformed connectome fingerprinting, as discussed in the main text.

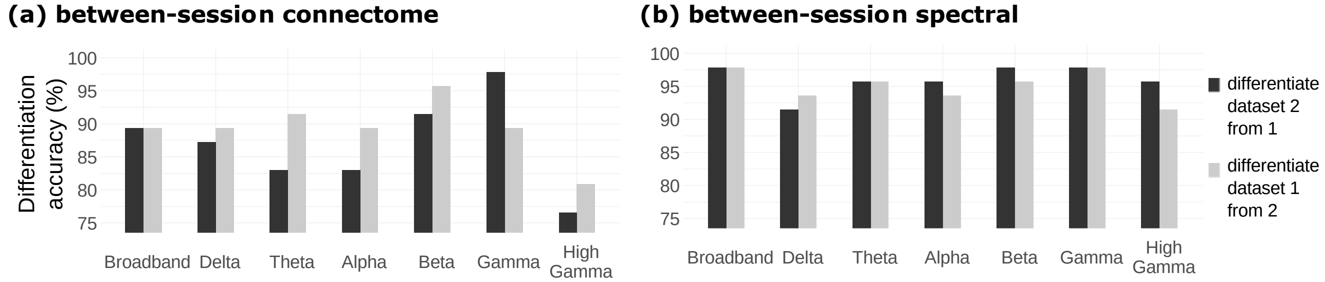

Supplementary Figure 7: Between-session differentiation accuracy

Results from MEG between-session fingerprinting. Differentiation accuracy for both (a) connectome and (b) spectral broadband and narrowband fingerprinting. The accuracy scores are reported for fingerprinting from dataset 1 to dataset 2 and vice-versa (see Methods). Source data are provided as a Source Data file.

Individuals cannot be differentiated from their respective imaging kernels

We verified that the within-session fingerprinting of individuals was not possible from empty-room data (i.e., with no participant under the MEG sensor array) processed through their respective imaging kernel of beamformer weights. Indeed, these latter are defined from individual anatomy and head position under the MEG sensor array, which may have been sufficient information to drive differentiation. We therefore ran the same fingerprinting pipeline on each session's empty-room data transformed through the corresponding individual's beamformer imaging kernel, which was identical for each of the within-session data segments used. Note that for the between-session challenges, the imaging kernels were adjusted to the respective individual head positions measured during each session. These analyses demonstrated that the imaging kernel information did not contribute substantially to MEG fingerprinting (overall performance was below 20% on average).

We also ran the MEG fingerprinting pipeline directly from the sensor data of the empty-room recordings, without transformation through individual imaging kernels, to assess the floor level of differentiation performances from non-brain data only. The data confirmed substantially lower levels of fingerprinting (<5% accuracy on average; see Supplementary Figure 8).

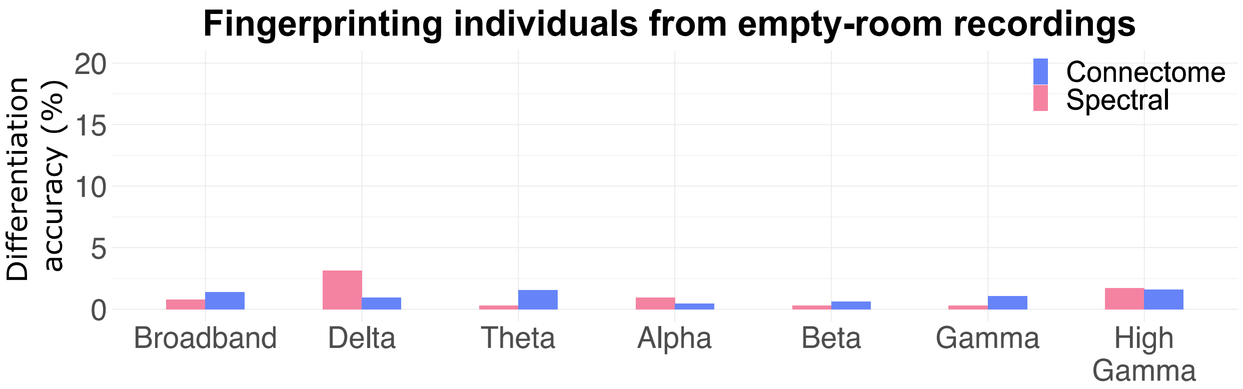

Supplementary Figure 8: Verification of failed fingerprinting from non-brain data (empty-room recordings)

Results for the empty-room sensor fingerprinting challenge. As expected, differentiation accuracies of connectome and spectral broadband and narrowband fingerprinting were substantially lower than from actual MEG data with individuals present. Source data are provided as a Source Data file.

Fingerprinting from scalp data only

We also performed MEG fingerprinting from individual sensor data, with no MEG source reconstruction to assess the added value of source modeling. We replicated the above MEG fingerprinting pipelines from the within-, within-shortened, and between- session analyses. Differentiation performances were less than with source modeling, especially from signal components in higher frequency bands and for the shortened challenges (see Supplementary Figure 9, 10, & 11). Yet for other signal components and longer durations, individuals remain differentiable from sensor-level data collected between sessions (>60% accuracy from broadband data), albeit with lower accuracy than when using MEG source transformations, which explicitly account for different head positions between sessions.

Taken together with the empty-room fingerprinting tests above, these results provide evidence that brain signals, not environmental conditions, were crucial for individual differentiation.

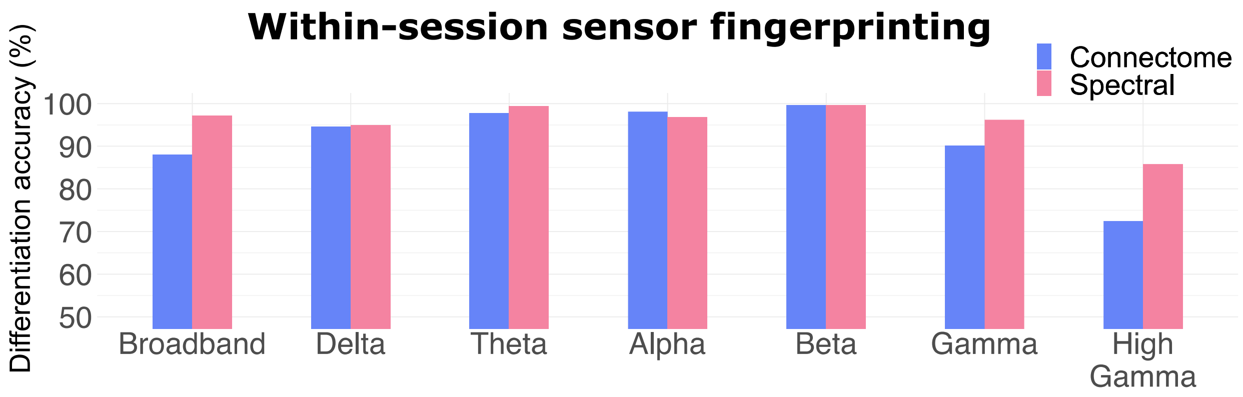

Supplementary Figure 9: Within-session differentiation from MEG sensor data (no source modeling)

Results from MEG sensor data in the within-session fingerprinting challenge. The differentiation accuracy statistics are shown for both connectome and spectral broadband and narrowband fingerprinting. The average accuracy scores are reported across differentiation from dataset-1 to dataset-2 and vice-versa (see Methods). Source data are provided as a Source Data file.

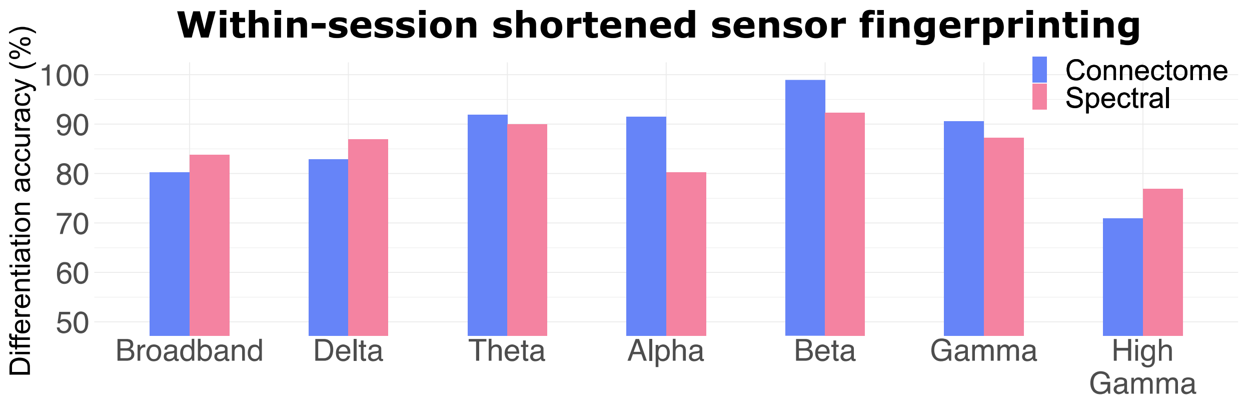

Supplementary Figure 10 Within-session differentiation from shortened (30-s) MEG sensor data (no source modeling)

Results from MEG sensor data in the within-session shortened fingerprinting challenge. The differentiation accuracy statistics are shown for both connectome and spectral broadband and narrowband fingerprinting. The average accuracy scores are reported across differentiation from all possible pairs of datasets (see Methods). Source data are provided as a Source Data file.

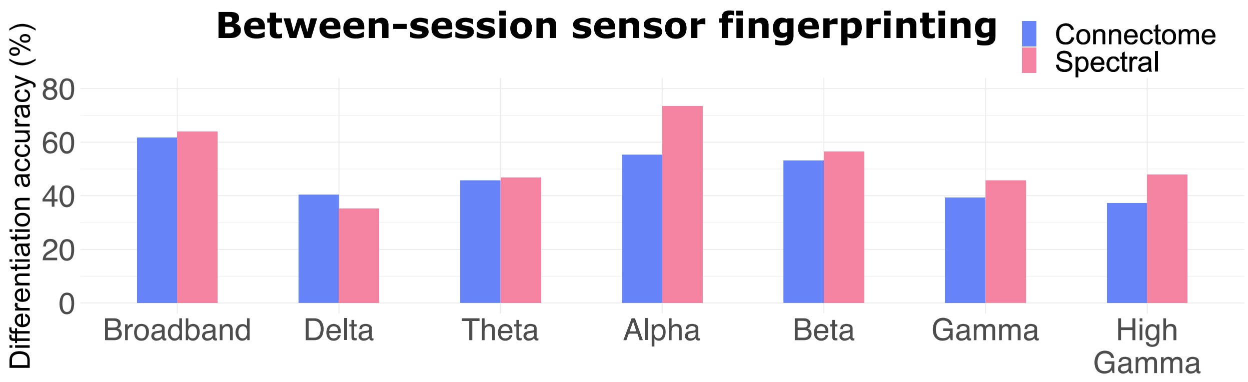

Supplementary Figure 11: Between-session differentiation from MEG sensor data (no source modeling)

Results from MEG sensor data in the between-session fingerprinting challenge. The differentiation accuracy statistics are shown for both connectome and spectral broadband and narrowband fingerprinting. The average accuracy scores are reported across differentiation from dataset-1 to dataset-2 and vice-versa (see Methods). Source data are provided as a Source Data file.

Salient neurophysiological features for fingerprinting

We reported in the main manuscript intraclass correlations (ICC) to determine which features contributed to individual differentiation the most. We also performed two additional analyses, deriving group consistency and differential power. These two metrics were proposed by Finn and colleagues (*2*) to identify the features which were the most consistent across their cohort, and the features which were the most consistent within individuals but different across participants, respectively (*2*). Differential power measures the empirical probability that a given feature is more likely to have a higher edgewise product vector across individuals than within the same individual. Taking the sum of the natural log of this probability across subjects yields differential power (*2*). The higher the differential power, the better a feature discriminates between individuals. Results for differential power are plotted in Figures S7 and S9. We found that the most discriminant connectome features were the visual and limbic networks across frequency bands, while the most discriminant spectral features remained along midline structures for fast oscillatory signal components. Overall, these results confirmed the ICC analysis results, with the addition of the contributions of spectral power in the beta and gamma band along the supplementary motor, motor, and somatosensory cortices.

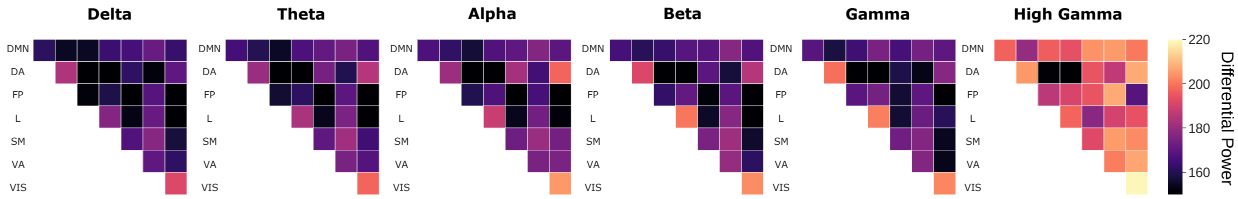

Supplementary Figure 12: Differential power connectome fingerprinting

Differential Power (DP) analysis for broadband connectome fingerprinting of the within-session dataset (see Figure 1). Mean DP plotted within frequency bands and per resting-state network as defined by (*3*): Default Mode Network (DMN), Dorsal Attention (DA), Frontal-Parietal (FP), Limbic (L), Somato-Motor (SM), Ventral Attention (VA), and Visual (VIS). The higher the DP, the more the corresponding functional connection was essential for fingerprinting. The outstanding connections determined by DP for fingerprinting were the Visual network across all frequency bands, and the Limbic network in the beta and gamma bands.

Group consistency reflects edges that are consistent across individuals. Group consistency was computed from the mean edgewise product vector across all subjects (*2*). Large values of group consistency highlight features that are consistent both within participants and across the cohort. Our analyses are shown Figures S8 and S10. The resulting most consistent connectome features remained along the diagonal of the FC matrix (i.e., connections within the same networks) specifically in the Dorsal Attention and Fronto-Parietal networks. The most consistent features for spectral fingerprinting were in the lower frequency bands, specifically in the lateral frontal cortices. This outcome was consistent with our ICC results (see Manuscript).

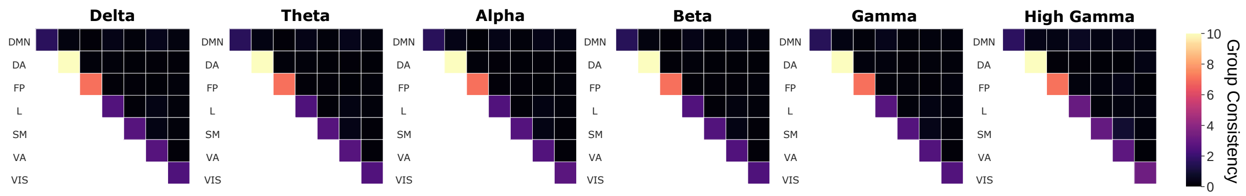

Supplementary Figure 13: Group consistency connectome fingerprinting

Group Consistency (GC) analysis for broadband connectome fingerprinting of the within-session dataset (see Figure 1). Mean GC plotted within frequency bands according to the labels from (*3*): Default Mode Network (DMN), Dorsal Attention (DA), Frontal-Parietal (FP), Limbic (L), Somato-Motor (SM), Ventral Attention (VA), and Visual (VIS). The higher the GC, the more consistent was a functional connection within an individual and across the cohort. The most consistent connections were those along the diagonal, specifically for the Dorsal Attention and Frontal-Parietal networks across all frequency bands.

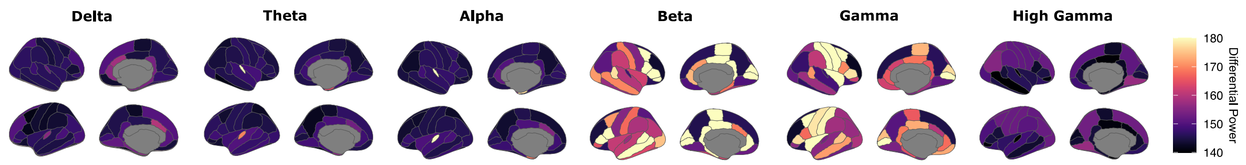

Supplementary Figure 14: Differential power spectral fingerprinting

Differential Power (DP) analysis for broadband spectral fingerprinting of the within-session dataset (see Figure 1). Mean DP plotted within frequency bands according to the Desikan-Killiany atlas (*4*).

The higher the DP, the more a given frequency band and ROI distinguished between individuals. The most characteristic regions and frequencies were medial structures for the beta band, and temporal and central regions for gamma band signals.

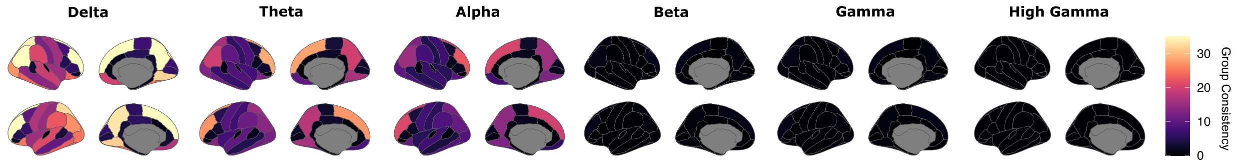

Supplementary Figure 15: Group consistency spectral fingerprinting

Group Consistency (GC) analyses for broadband spectral fingerprinting of the within-session dataset (see Figure 1). Mean GC plotted within frequency bands according to the Desikan-Killiany atlas (*4*). The higher the GC, the more a given frequency band and ROI remained consistent within individuals and across the cohort. The most stable frequencies were the lower bands (delta and theta) and the most consistent regions across individuals were lateral frontal areas.

Partial Least Squares (PLS) analysis

We tested whether differences in resting-state neurophysiological signals related to meaningful demographic features using an exploratory Partial Least Squares (PLS) analysis. PLS is a multivariate statistical method that relates two data matrices based on latent variables (LV) that explain the highest covariance between the two datasets. Here, our two datasets consist of a demographic matrix (i.e., age, gender, handedness, and clinical status) and a neurophysiological data matrix (i.e., spectral power or functional connectome). Latent variables (which explain the most covariance between both matrices), and their corresponding variance explained are plotted in Supplementary Figure 16. Significance of each latent variable was assessed via permutation tests. Permuting the rows of the data allowed us to compute an associate p-value for each latent variable (see Manuscript). We chose to explore the first significant latent variable which explained the most variance for each neurophysiological signal feature (i.e., the first component for connectomes and spectral data). The resulting weights associated to the latent neural and demographic components are depicted Figure 5 along with their bootstrapped ratios. These results corroborate how neurophysiological signals at rest, in addition to differentiating individuals, carry meaningful information about participant demographics.

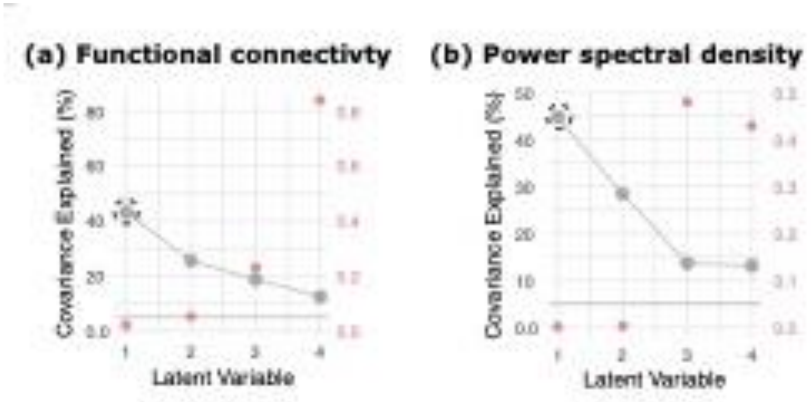

Supplementary Figure 16: PLS latent variables

Results for the PLS analysis conducted for both **(a)** connectome and **(b)** spectral fingerprinting features. Each plot depicts the latent components obtained for each of the PLS analyses, their corresponding variance explained, and permuted *p*-value (right axis). P-values were determined based on permutations of the data to obtain a null distribution. One significant latent variable explained 43.1% of the variance for connectome fingerprinting (*p* = 0.021) and two latent variables explained 44.7% *(p = 0.001)* and 28.3% *(p = 0.003)* of the variance for spectral fingerprinting, respectively. We explored in the main Manuscript only the first significant component for each method (i.e., the circled component). Source data are provided as a Source Data file.

Supplementary References

1. E. Amico, J. Goñi, The quest for identifiability in human functional connectomes. *Sci. Rep.* **8**, 8254 (2018).

2. E. S. Finn, X. Shen, D. Scheinost, M. D. Rosenberg, J. Huang, M. M. Chun, X. Papademetris, R. T. Constable, Functional connectome fingerprinting: identifying individuals using patterns of brain connectivity. *Nat. Neurosci.* **18**, 1664–1671 (2015).

3. B. T. Yeo, Thomas, F. M. Krienen, J. Sepulcre, M. R. Sabuncu, D. Lashkari, M. Hollinshead, J. L. Roffman, J. W. Smoller, L. Zöllei, J. R. Polimeni, B. Fischl, H. Liu, R. L. Buckner, The organization of the human cerebral cortex estimated by intrinsic functional connectivity. *J. Neurophysiol.* **106**, 1125–1165 (2011).

4. R. S. Desikan, F. Ségonne, B. Fischl, B. T. Quinn, B. C. Dickerson, D. Blacker, R. L. Buckner, A. M. Dale, R. P. Maguire, B. T. Hyman, M. S. Albert, R. J. Killiany, An automated labeling system for subdividing the human cerebral cortex on MRI scans into gyral based regions of interest. *NeuroImage*. **31**, 968–980 (2006).
