# Supplementary material for: Brief segments of neurophysiological activity enable individual differentiation
Source: Nat Commun. 2021 Sep 29;12:5713. doi: 10.1038/s41467-021-25895-8 (PMC8481307; doi:10.1038/s41467-021-25895-8)
Supplement: Supplementary file 3 — Reporting summary [file 41467_2021_25895_MOESM3_ESM.pdf]

nature portfolio

Corresponding author(s): Dr. Sylvain Baillet
Dr. Bratislav Misic

Last updated by author(s): Aug 11, 2021

Reporting Summary

Nature Portfolio wishes to improve the reproducibility of the work that we publish. This form provides structure for consistency and transparency in reporting. For further information on Nature Portfolio policies, see our Editorial Policies and the Editorial Policy Checklist.

Statistics

For all statistical analyses, confirm that the following items are present in the figure legend, table legend, main text, or Methods section.

n/a	Confirmed	
☐	☒	The exact sample size (n) for each experimental group/condition, given as a discrete number and unit of measurement
☐	☒	A statement on whether measurements were taken from distinct samples or whether the same sample was measured repeatedly
☐	☒	The statistical test(s) used AND whether they are one- or two-sided<br>*Only common tests should be described solely by name; describe more complex techniques in the Methods section.*
☒	☐	A description of all covariates tested
☒	☐	A description of any assumptions or corrections, such as tests of normality and adjustment for multiple comparisons
☐	☒	A full description of the statistical parameters including central tendency (e.g. means) or other basic estimates (e.g. regression coefficient) AND variation (e.g. standard deviation) or associated estimates of uncertainty (e.g. confidence intervals)
☐	☒	For null hypothesis testing, the test statistic (e.g. F, t, r) with confidence intervals, effect sizes, degrees of freedom and P value noted<br>*Give P values as exact values whenever suitable.*
☒	☐	For Bayesian analysis, information on the choice of priors and Markov chain Monte Carlo settings
☒	☐	For hierarchical and complex designs, identification of the appropriate level for tests and full reporting of outcomes
☐	☒	Estimates of effect sizes (e.g. Cohen's d, Pearson's r), indicating how they were calculated

Our web collection on statistics for biologists contains articles on many of the points above.

Software and code

Policy information about availability of computer code

Data collection	No software was used to collect data. All data was obtained from an open dataset (OMEGA) see Methods and Data Availability statement
Data analysis	Preprocessing was completed in MATLAB2017b, and brainstorm Version Oct12-2018. The connectome and spectral features were exported to Python (3.7.6) for subsequent fingerprinting analyses. All codes for including preprocessing and data analysis can be found on the project's GitHub. Rotman-Baycrest PLS toolbox V. 6.13 was used for the multivariate statistical analyses.

For manuscripts utilizing custom algorithms or software that are central to the research but not yet described in published literature, software must be made available to editors and reviewers. We strongly encourage code deposition in a community repository (e.g. GitHub). See the Nature Portfolio guidelines for submitting code & software for further information.

Data

Policy information about availability of data

All manuscripts must include a data availability statement. This statement should provide the following information, where applicable:

- Accession codes, unique identifiers, or web links for publicly available datasets
- A description of any restrictions on data availability
- For clinical datasets or third party data, please ensure that the statement adheres to our policy

Resting-state recording were obtained from the OMEGA database. Raw resting-state MEG recordings can be accessed by requesting the data (https://www.mcgill.ca/bic/omega-registration). The power spectra and connectomes derived from the preprocessed OMEGA samples and used to differentiate individuals in the present study are available from the corresponding author on reasonable request. Source data are provided with this paper.

Field-specific reporting

Please select the one below that is the best fit for your research. If you are not sure, read the appropriate sections before making your selection.

☐ Life sciences ☒ Behavioural & social sciences ☐ Ecological, evolutionary & environmental sciences

For a reference copy of the document with all sections, see nature.com/documents/nr-reporting-summary-flat.pdf

Behavioural & social sciences study design

All studies must disclose on these points even when the disclosure is negative.

Study description	The study acquired quantitative data from participants using neuroimaging (MEG: magnetoencephalography.)
Research sample	We obtained our data from the OMEGA repository (Open MEG Archives). In brief, we analyzed MEG resting-state data from 158 unrelated OMEGA participants (77 Females, 31.9 ± 14.7 years old). Recordings were approximately 5-min long. Supplementary Table 1 provides details of scanning procedures and Supplementary Table 2 reports demographics. Most participants (n= 130) were healthy controls, and a small subset were from a clinical population (i.e., chronic pain and ADHD).
Sampling strategy	Data sample size was determined by the amount of preexisting open data available in the OMEGA repository Since fingerprinting does not require statistical inferences, we did not predetermine our sample size before our analyses. We simply relied on the data that were available through OMEGA.
Data collection	Resting state data were collected with a MEG system (275 channels whole-head CTF; Port Coquitlam, British Columbia, Canada). The sampling rate was 2400 Hz, with an antialiasing filter applied at 600 Hz cut-off, and built-in third-order spatial gradient noise cancellation (see 6 for details on data acquisition). Only the participant and experimenters were present during data collection. Experimenters were blind to the hypotheses and questions concerning the present study.
Timing	All data available from the OMEGA repository (as of Sep 2018) were downloaded. The OMEGA repository continuously collects participants that provided consent to contribute to OMEGA. Our sample consisted of data collected from Jan 2015 to Jan 2018
Data exclusions	No data was excluded from our analyses.
Non-participation	No participants dropped out.
Randomization	There were no experimental groups, no randomization required.

Reporting for specific materials, systems and methods

We require information from authors about some types of materials, experimental systems and methods used in many studies. Here, indicate whether each material, system or method listed is relevant to your study. If you are not sure if a list item applies to your research, read the appropriate section before selecting a response.

Materials & experimental systems

n/a	Involved in the study	
☒	☐	Antibodies
☒	☐	Eukaryotic cell lines
☒	☐	Palaeontology and archaeology
☒	☐	Animals and other organisms
☐	☒	Human research participants
☒	☐	Clinical data
☒	☐	Dual use research of concern

Methods

n/a	Involved in the study	
☒	☐	ChIP-seq
☒	☐	Flow cytometry
☒	☐	MRI-based neuroimaging

Human research participants

Policy information about studies involving human research participants

Population characteristics	See above.
Recruitment	Data was obtained from the OMEGA data repository. Convenience sampling was used to recruit participants. Our results are limited, as by all other studies conducting convenience sampling, to a highly educated, western, and generally healthy population. As we study individual differentiation, we do not expect our results to be biased by our sampling methods.
Ethics oversight	We followed ethical procedures from the research ethics board of the Montreal Neurological Institute .

Note that full information on the approval of the study protocol must also be provided in the manuscript.
